# All for All

**DOI:** 10.1371/journal.pbio.0040198

**Published:** 2006-06-13

**Authors:** John Sulston

## Abstract

A review of Michael Ashburner's book *Won for All: How the* Drosophila *Genome Was Sequenced*.

**Figure:**
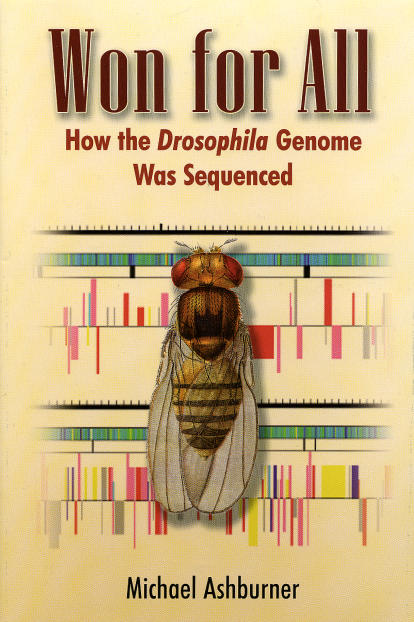


In this small and charming book, *Won for All*, Michael Ashburner gives us a glittering account of the sequencing of the Drosophila genome by a public–private partnership between government-funded laboratories and Celera Genomics. He portrays both the working life and the good life of science, with neat character sketches set off by Lewis Miller's excellent portraits. Michael's flair for detail and inveterate name-dropping, albeit of restaurants rather than people, lends itself nicely to re-creating the time and place of key events in this collaboration. The original fast-paced manuscript, which I liked so well when I first saw a draft in 2001, has been updated and provided with extensive footnotes that inform without interrupting the narrative. Technical background is given in two excellent postscripts: a fly primer from Scott Hawley, and an overview of fly functional genomics from Ethan Bier.

Michael and I have been orbiting around Cambridge (the original one, as he puts it) for many years. Our paths intersect occasionally and pleasantly, but never so forcibly as over genomics—in keeping with Maurice Wilkins' remark to Horace Judson that: “DNA, you know, is Midas' gold. Everyone who touches it goes mad” [ 1]. It has indeed proved so in the age of sequencing genomes.

One of the emergent themes of the book is competition between animal model organisms, which was intense from the 1960s to the 1990s. Flies, worms, molluscs, mice, and men: each had its own set of dedicated followers, and each had strengths and weaknesses in the common goal of understanding the biology of animals and humans in particular. At a slightly greater distance, there was competition with micro-organisms and plants for understanding life in general.

Compared with the nematode worm, on which I worked, the fly was the doyen, with half a century of intensive work behind it, 10-fold more researchers, a rich morphology of wings and legs and bristles, and a huge range of sophisticated genetic techniques. The worm was a shy newcomer, kicked into excitement by Sydney Brenner, who chose it for its small size and rapid growth. Never mind the fly's sophistication; for those of us who came early the worm was a sandpit to play in: anything we did was novel because so little had been done before.

With the advent of molecular cloning in the 1970s, developmental biology entered a new and thrilling phase. No longer were experiments scored only by visible outcomes; now the aim was to isolate and study the genes controlling these tangible phenomena. There were triumphs: for example, homeobox genes controlling development in the fly, and cell-death genes found in the worm. And now the fly displayed anew one of its long-known attributes: the polytene chromosomes in the salivary glands. Composed of multiple aligned copies of the fly's DNA, they provide an extraordinary pocket map of its genome, gratis. The polytenes were so advantageous to gene hunters that attempts were made to find a genetic modification of the worm that would produce something comparable, to no avail. However, this initial disadvantage soon turned to the advantage of the worm researchers, because we were forced to construct our own physical map from cloned fragments of worm DNA. Clumsy and incomplete at first, the map grew into a full coverage of the genome. We arrayed our clones on membranes and proudly announced our artificial polytenes. And the arrays were more than polytenes: not only a map, but actual fragments that could be shipped around the world and used directly in people's experiments.

More was to come. The physical map allowed the worm an early start on genome sequencing, long before automation was sufficiently advanced to tackle the whole thing. Our sequence was publicly released as we went along, and soon, as researchers isolated individual genes from human and other organisms, they more often found a match to worm than anything else. People were drawn to the worm, and it prospered, increasing pressure on the fly community to start their own genome project.

I had nothing to do with the fly scientifically, but its genome made a big impact on me through the Human Genome Project. Michael disarmingly suggests that it's a mystery why Celera would sequence the fly for no financial return. The reason, of course, was that it provided a doable and very public advertisement for the human genome sequence, which was Celera's real business. It meant that Gerry Rubin, as leader of the fly sequencing project, could be persuaded to speak in support of the company at US congressional hearings that discussed appropriations for the public sequencing effort. Celera's fly sequence was indeed “won for all,” but for the human it would be a different story, as those who searched for public–private partnership discovered in December 1999 [ 2]. And where human sequence went, there went everything. Had the human genome sequencing project gone private, the public databases, which are one of the most important foundations of modern biology, would have withered and been replaced by a corporate entity under the control not of its users but of its shareholders.

Gerry was the fly hero, doing what he had to do to get his organism sequenced. But despite Scott Hawley's opening words, Michael is a hero for us all. Celera initially posted the fly sequence with copyright restrictions, against all previous agreements, and it was Michael who ensured that the restrictions were removed. Later, he was prominent in supporting free release of the human sequence. Today, in his development of FlyBase and Gene Ontology, and in his championing of open-access publication, he continues as a giant of public-domain science.

The marvellous outcome is that the worm, the fly, the human, and hundreds of other genomes have all been *won for all*. The rivalries have been transformed into a new wholeness of biology, united through its common history written in the language of the genomes.
